# Meteorological Report for May

**Published:** 1879-06

**Authors:** 


### METEOROLOGICAL REPORT FOR MAY, 1879.

| RAIN. DATE | RAIN. DAY. | Mean daily Barometer. | Mean daily Thermometer | Mean daily Humidity. | Maximum Thermometer | Minimum Thermometer | Prevailing direction. |
|---|---|---|---|---|---|---|---|
| 1 | 21 | 29.839 | 61.7 | 64.3 | 75 | 55 | N. W. |
| 2 | 0 | 29 997 | 59.2 | 29.3 | 66 | 49 | N. W. |
| 3 | 0 | 29.983 | 63.0 | 28.7 | 73 | 49 | S. E. |
| 4 | 0 | 29.868 | 72.2 | 46.0 | 81 | 55 | W. |
| 5 | 32 | 29.883 | 71.0 | 69.3 | 79 | 63 | N. W. |
| 6 | 0 | 30 005 | 66.7 | 60.3 | 74 | 62 | N. W. |
| 7 | 37 | 30.045 | 61.0 | 77.7 | 68 | 55 | N. E. |
| 8 | 0 | 30.112 | 65.2 | 64.3 | 72 | 53 | N. E. |
| 9 | 0 | 30 241 | 64 2 | 44.0 | 71 | 55 | N. E. |
| 10 | 0 | 30.197 | 63.7 | 31 0 | 72 | 50 | N. E. |
| 11 | 0 | 30.062 | 67.0 | 57.3 | 77 | 53 | N. E. |
| 12 | 0 | 29.994 | 68.2 | 67.7 | 75 | 63 | N. E, |
| 13 | 18 | 29 962 | 67.7 | 81.7 | 76 | 64 | E. |
| 14 | 1.42 | 29 865 | 66.7 | 86.0 | 74 | 64 | E. |
| 15 | 0 | 29.742 | 70.2 | 66.7 | 78 | 63 | S. E. |
| 16 | 08 | 29.819 | 67.5 | 54.5 | 77 | 60 | S. E. |
| 17 | 1.34 | 29 912 | 64.5 | 84.7 | 68 | 61 | N. |
| 18 | 24 | 29.906 | 69.0 | 71.7 | 79 | 59 | N. |
| 19 | 0 | 28.867 | 74.5 | 54.3 | 82 | 62 | N. W. |
| 20 | 0 | 29.802 | 77.5 | 49.0 | 85 | 65 | N. W. |
| 21 | 0 | 29.792 | 78.5 | 53.0 | 87 | 67 | E. |
| 22 | 0 | 29.919 | 76.7 | 52.7 | 88 | 68 | N. E. |
| 23 | 0 | 30.182 | 69.2 | 55.7 | 76 | 66 | N. E. |
| 24 | 0 | 30.235 | 69.5 | 54.7 | 75 | 56 | S. E. |
| 25 | 0 | 30.069 | 77.7 | 45.3 | 83 | 62 | S. W. |
| 26 | 0 | 30.019 | 80.2 | 46.3 | 86 | 68 | S. W. |
| 27 | 0 | 30.099 | 79.5 | 49.3 | 90 | 70 | W. |
| 28 | 0 | 30.058 | 81.5 | 47.7 | 91 | 66 | S. E. |
| 29 | 0 | 30.022 | 79.5 | 39.7 | 89 | 69 | E. |
| 30 | 0 | 30 035 | 79.5 | 45.7 | 86 | 68 | E. |
| 31 | 0 | 30.041 | 81.0 | 43.3 | 88 | 69 | S. E. |
| Monthly Means. | | 29.988 | 70.8 | 55.5 | 78.7 | 60.9 | N. E. |

Highest Barometer, 30.345 on the 24th.
Lowest Barometer, 29.664, on the 15th.
Monthly range of Barometer, 0.681.
Highest Temperature, 91°, on 28th. Lowest Temperature, 49°, 2d&3d.
Monthly range of Temperature, 42°.
Greatest daily range of Temperature, 26°, on the 4th.
Least daily range of Temperature, 7°, on the 17th.
Mean of Maximum Temperatures, 78°7.
Mean of Minimum Temperatures, 60°9.
Mean daily range of Temperature, 17°8. Total rainfall, 4.16 inches.
Prevailing wind, Northeast. Total movement of wind, 60.80 miles.
Maximum velocity of wind and direction, 22 miles per hour, Northeast, on the 9th and 11th.
*Number of Foggy days, none. No. Cloudy days on which rain fell, 6.
Number of cloudy days on which no rain fell, 4.
Total number of days on which rain fell, 9.

H. HALL, Corp'l. Signal Service U.S.

Atlanta, Ga., May, 1879.

